# Moyamoya Disease and Syndrome in Adults: A Report of Four Cases

**DOI:** 10.7759/cureus.85357

**Published:** 2025-06-04

**Authors:** Ghizlane Es-sayeh, Siham Bouchal, Y Lamrani, Mustapha Maaroufi, Mohammed Faouzi Belahsen

**Affiliations:** 1 Neurology, Hassan II University Hospital, Fez, MAR; 2 Radiology, Hassan II University Hospital, Fez, MAR

**Keywords:** digital subtraction angiography, hemorrhagic stroke, hyperthyroidism, ischemic stroke, moyamoya disease and syndrome, sjögren’s syndrome

## Abstract

Moyamoya disease and syndrome is a rare angiogenic disorder characterized by progressive narrowing of the distal internal carotid artery and proximal segments of the middle and anterior cerebral arteries, leading to the formation of replacement vessels to compensate for reduced cerebral perfusion downstream of the stenosis. Its etiology remains poorly understood, and its most serious consequences are ischemic or hemorrhagic stroke. It may be primary or secondary. We present four cases of Moyamoya disease and syndrome, including three women and one man, with ages of 42, 44, 46, and 45 years, respectively. Three patients presented with ischemic stroke, and one patient experienced multiple ischemic and hemorrhagic cerebrovascular events. All four had undergone emergency cerebral CT and/or MRI, but the diagnosis was made by CT angiography and cerebral digital subtraction angiography. All four patients received medical treatment with calcium channel blockers and antiplatelet agents, with rehabilitation, and none underwent revascularization. The etiological investigation revealed Sjögren’s syndrome in one patient, hyperthyroidism in another, and no abnormalities in the remaining cases. Through these four observations, we discuss the clinical presentations, diagnosis, and therapeutic features of this rare condition.

## Introduction

Moyamoya disease and syndrome is a rare but serious disorder of the cerebral vessels, characterized by progressive steno-occlusion of the intracranial arteries. This stenosis results in reduced cerebral blood flow, thereby increasing the risk of ischemia. Strokes generally result from the formation of parenchymal, perforating, and leptomeningeal collaterals from neovascularization. These collateral vessels, visible on digital subtraction angiography, present a distinctive appearance by forming a dense "smoke cloud"-like network, characteristic of Moyamoya disease [1]. Diagnosis relies mainly on brain imaging, particularly digital subtraction angiography, to visualize these vascular anomalies. In children and adolescents, ischemia is more common, while in adults, intracranial bleeding is more common (19.1% to 42.3%), with fewer cases of transient ischemic attack or cerebral infarction (57.7% to 70.0%) [2]. There are two peaks in incidence: one in children around the age of 5 and another in adults around the age of 40 [1]. Progression can be gradual, with intermittent symptoms, or more acute, resulting in rapid neurological and cognitive decline.

## Case presentation

Case 1

**Figure 1:**
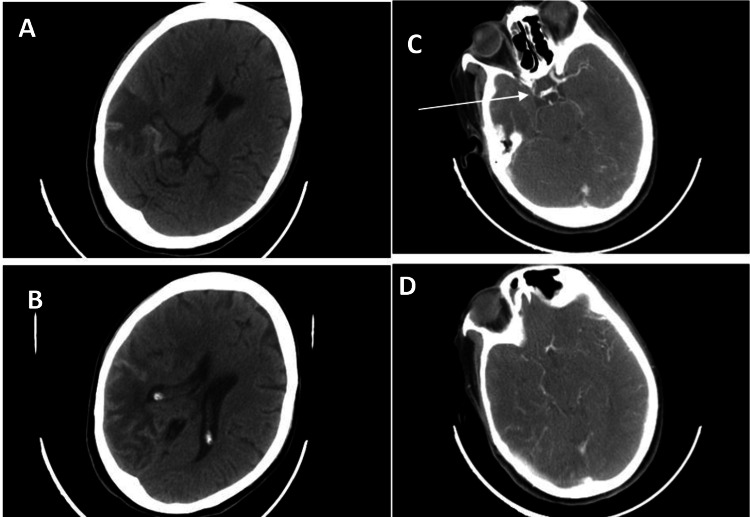
(A, B) CT scan: right temporo-parietal hypodense area with peripheral cortical hyperdensity, suggestive of a right middle cerebral ischemic stroke with resolving hemorrhagic transformation. (C, D) CT angiography: reduction in the caliber of the right carotid artery, stopping at the level of the intracranial internal carotid artery, with the absence of visualization of the proximal segments of the right anterior and middle cerebral arteries CT: computed tomography

**Figure 2:**
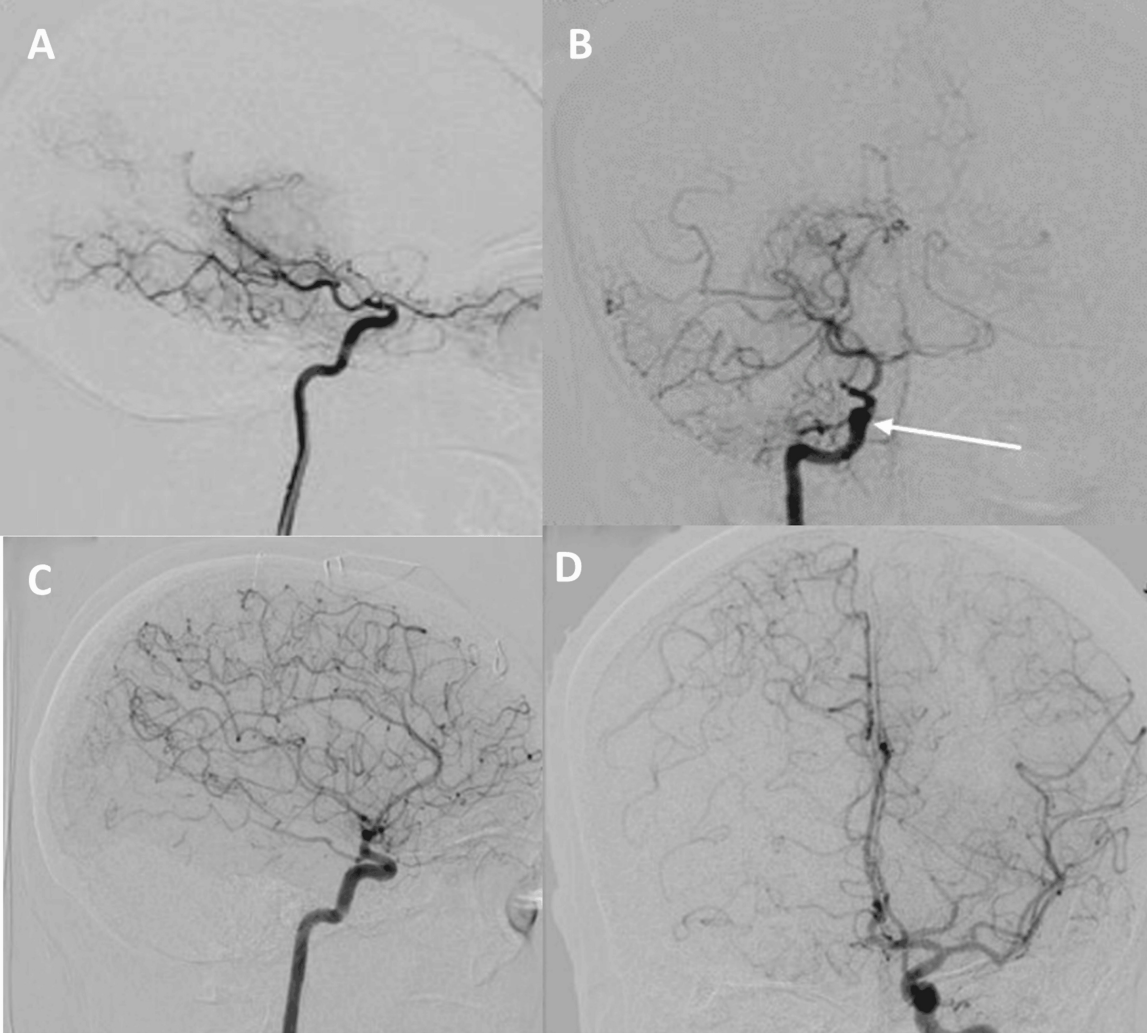
Common and internal right carotid digital subtraction angiography lateral view (A) and frontal view (B) showing stenosis of the right internal carotid artery along its entire length and a total intracranial terminal carotid stenosis. (C, D) Normal appearance of the left internal carotid artery and left anterior circulation. Complete collateral circulation of the right anterior circulation through the anterior communicating artery, with slight asymmetry in the circulatory dynamics between the right and left sides

A 42-year-old female patient with no medical history was admitted for a sudden onset of left weakness, accompanied by facial asymmetry. Neurological examination revealed left hemiplegia with central facial paralysis and left homonymous hemianopia. Cerebral CT showed a right middle cerebral artery infarct (Figure 1), and CT angiography showed reduced caliber of the right internal carotid artery and lack of visualization of the proximal segments of the right anterior and middle cerebral arteries (Figure 1). Arteriography revealed stenosis of the right distal internal carotid artery, which is occluded in the intracavernous carotid artery, with absence of opacification of the A1 segment of the right anterior cerebral artery and the right middle cerebral artery, with an abnormal vascular network (Moyamoya vessels) (Figure 2). The inflammatory and immunological workup was negative, and the CSF study and thoraco-abdominopelvic CT scan showed no abnormalities (Table 1). The diagnosis of Moyamoya disease was confirmed. Platelet antiaggregant and rehabilitation were started. Clinical improvement was noted with partial recovery of the motor deficit. No ischemic or hemorrhagic recurrences were observed during follow-up.

**Table 1:** Biological profiles of the four Moyamoya patients ESR: erythrocyte sedimentation rate, CRP: C-reactive protein, CSF: cerebrospinal fluid, CT: computed tomography, TSH: thyroid-stimulating hormone

Case	Inflammatory markers	Immunological workup	CSF analysis	Other notable tests	Associated conditions
1	Normal	Negative	Normal	Normal thoraco-abdominopelvic CT scan	None
2	Normal	Negative	Normal	Normal thoraco-abdominopelvic CT scan	None
3	Elevated (ESR, CRP)	Positive (Sjögren's syndrome: salivary biopsy with FS=4; dry eye; low salivary flow)	Isolated hyperproteinorachia (1.2 g/L)	Diabetes, hypertension	Secondary Moyamoya syndrome (Sjögren’s syndrome)
4	Normal	Negative	Normal	Hyperthyroidism (TSH ↓, FT4 ↑, FT3 ↑), multinodular goiter	Secondary Moyamoya syndrome (toxic multinodular goiter)

Case 2

**Figure 3:**
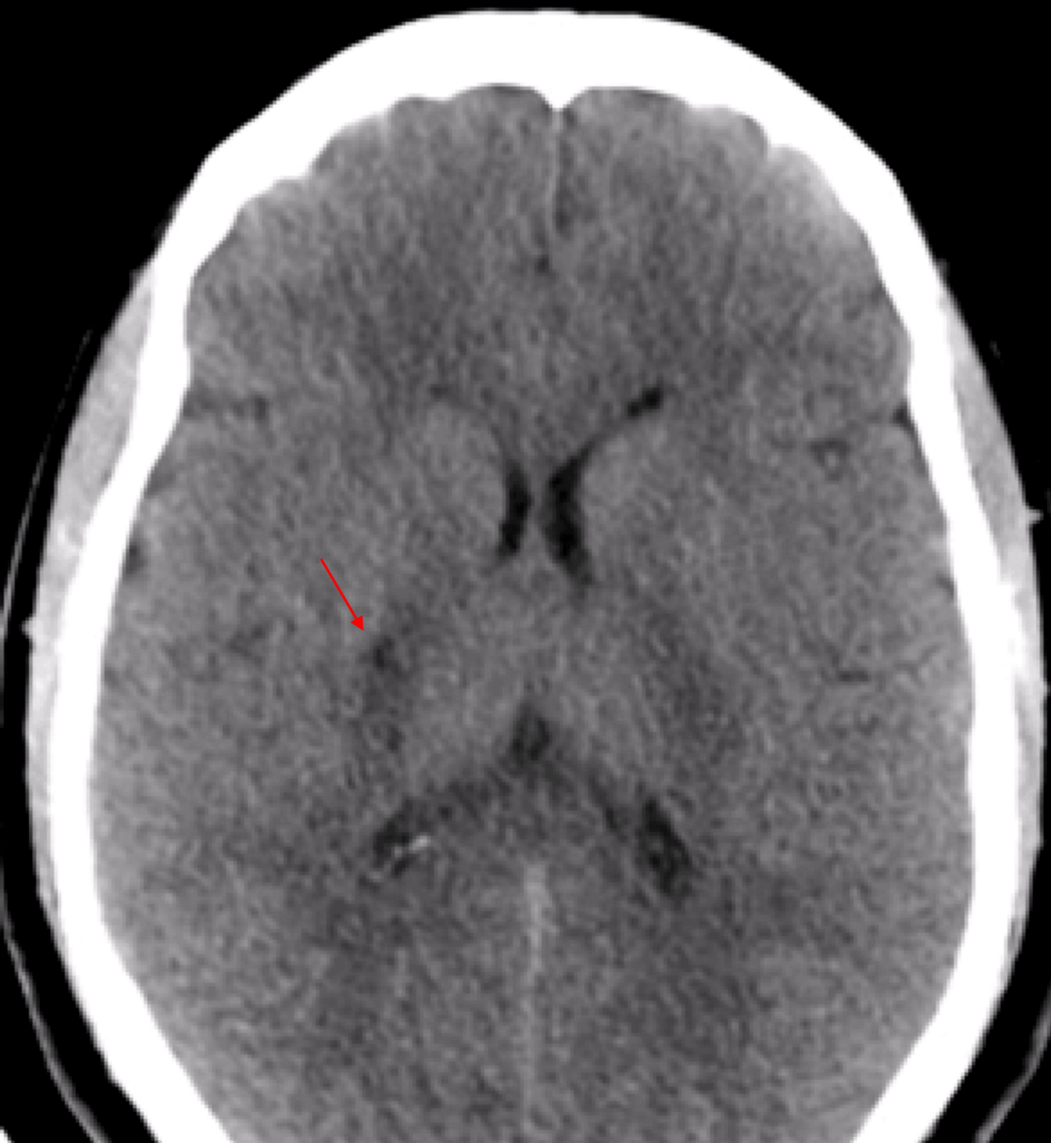
Cerebral CT scan: right capsulo-lenticular hypodensity (red arrow), indicative of a right ischemic stroke CT: computed tomography

**Figure 4:**
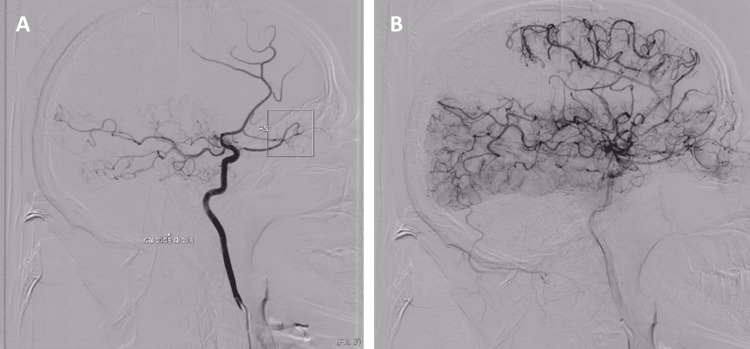
Digital subtraction angiography: (A) presence of a stenosis at the proximal portions of M1 of the right MCA and A1 of the ipsilateral ACA, presence of stenosis of A1 of the left ACA, and (B) reestablishment of circulation in the anterior territories from the MCA and posterior arteries MCA: middle cerebral artery, ACA: anterior cerebral artery

A 44-year-old man with no medical history presented to the emergency department for sudden left weakness accompanied by a left facial asymmetry, which completely resolved after five hours. He presented three days ago with the same symptoms, which completely resolved after 30 minutes. Neurological examination at admission was normal, with blood glucose and blood pressure within normal values. Cerebral CT showed hypodensity in the right internal capsule, extending to the homolateral lenticular nucleus (Figure 3). Cerebral CT angiography revealed a stenosis of the M1 portion of the right middle cerebral artery and stenosis in the vertebral arteries. Digital subtraction angiography confirmed stenosis of the proximal portion of the right anterior and middle cerebral arteries, with an abnormal vascular network from the middle and posterior cerebral arteries (Figure 4). The inflammatory and immunological workup was negative, and the CSF study and thoraco-abdominopelvic CT scan showed no abnormalities (Table 1). The patient was treated with antiplatelet agents and has not presented any ischemic or hemorrhagic recurrence to date.

Case 3

**Figure 5:**
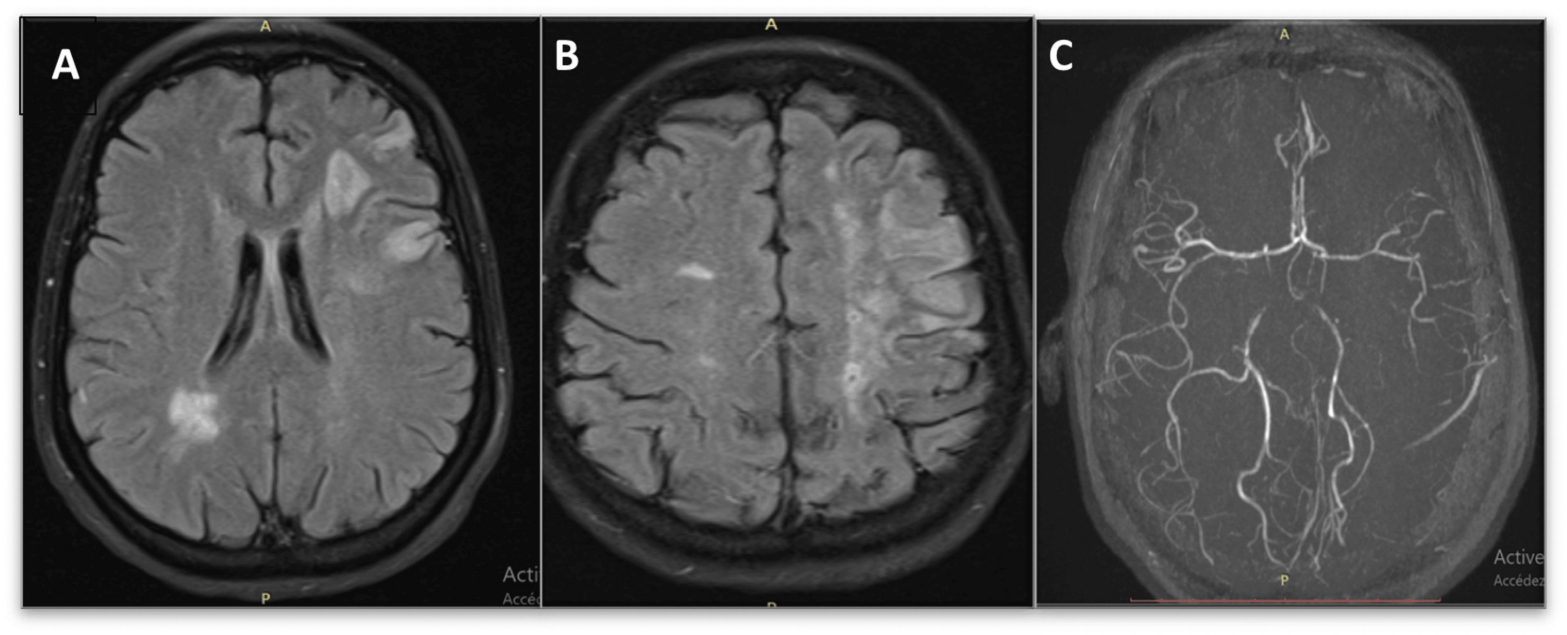
MRI with axial slices: (A, B) FLAIR sequence showing ischemic strokes of different ages. (C) MRA: irregular appearance of the intracranial arteries, particularly the left carotid artery MRI: magnetic resonance imaging, FLAIR: fluid-attenuated inversion recovery, MRA: magnetic resonance angiography

**Figure 6:**
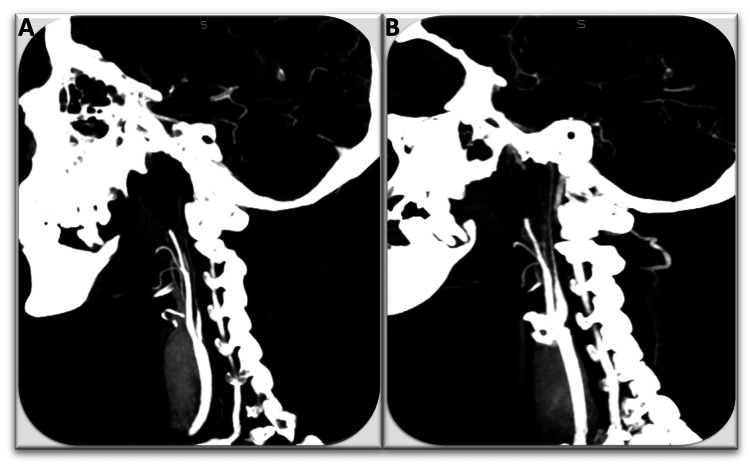
CT angiography of the CAS: filiform appearance of the internal carotid arteries. (A) right carotid and (B) left carotid CT: computed tomography, CAS: carotid artery stenosis

**Figure 7:**
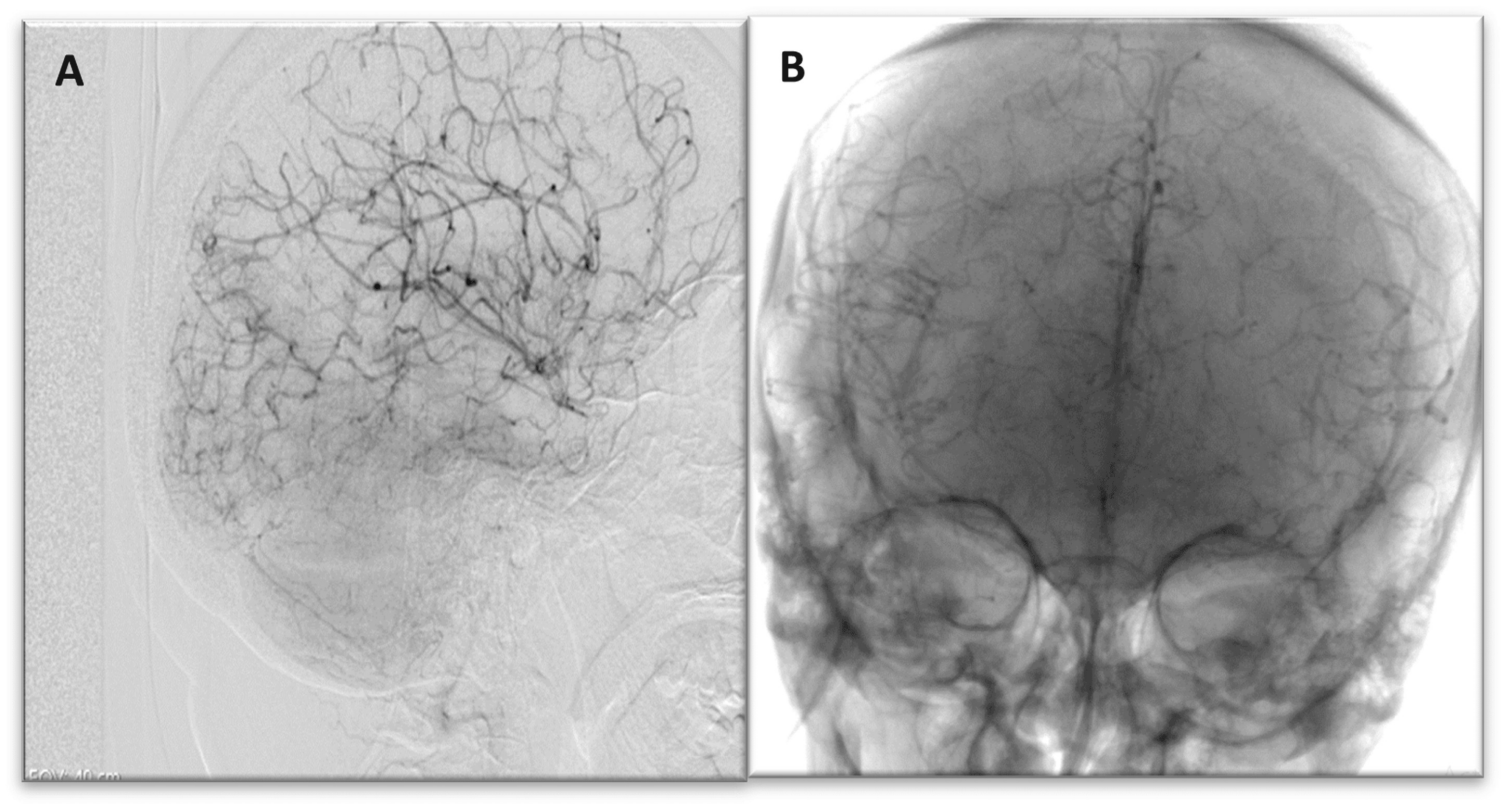
Digital subtraction angiography: (A) occlusion of the terminal portions of both internal carotid arteries with (B) collateral circulation, resulting in a Moyamoya appearance

A 46-year-old woman who had been followed for two years for hypertension, type 2 diabetes, and inflammatory arthralgias. She was admitted to the emergency department for sudden right weakness accompanied by mutism. On clinical examination, the NIHSS score was 13. Cerebral MRI revealed several cerebral infarcts of different locations and ages (Figure 5). Cerebral CT angiography showed a thread-like appearance of both internal carotid arteries (Figure 6). Digital subtraction angiography revealed occlusion of the terminals of both internal carotid arteries, with a typical Moyamoya supply network (Figure 7). Biological tests revealed an inflammatory blood syndrome, and CSF showed isolated hyperproteinorachia at 1.2 g/L. Salivary biopsy showed lymphocytic sialadenitis with a focus score of 4. Ophthalmological examination revealed a dry eye syndrome, with a salivary flow of 1.2 ml after 15 minutes (Table 1). The diagnosis was Moyamoya syndrome secondary to Sjögren's syndrome, complicated by ischemic and hemorrhagic strokes. Initial treatment consisted of dual therapy with antiplatelet and rituximab. Long-term follow-up showed complete recovery of motor deficit, although vascular dementia appeared. No subsequent ischemic or hemorrhagic events were observed.

Case 4

**Figure 8:**
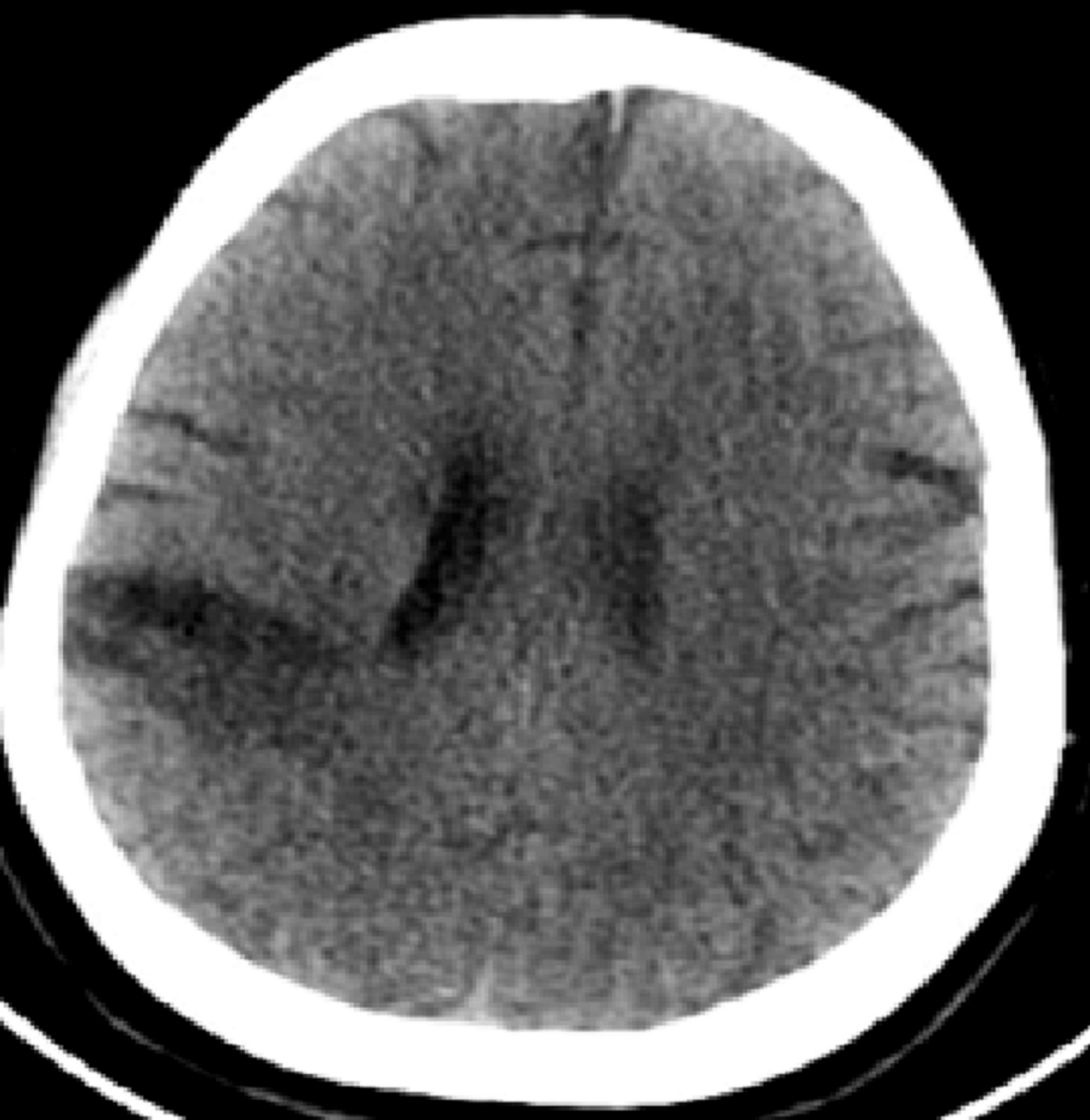
Cerebral CT scan: right posterior fronto-cortical subcortical hypodense area involving the territory of the right MCA, suggestive of late ischemic changes CT: computed tomography, MCA: middle cerebral artery

**Figure 9:**
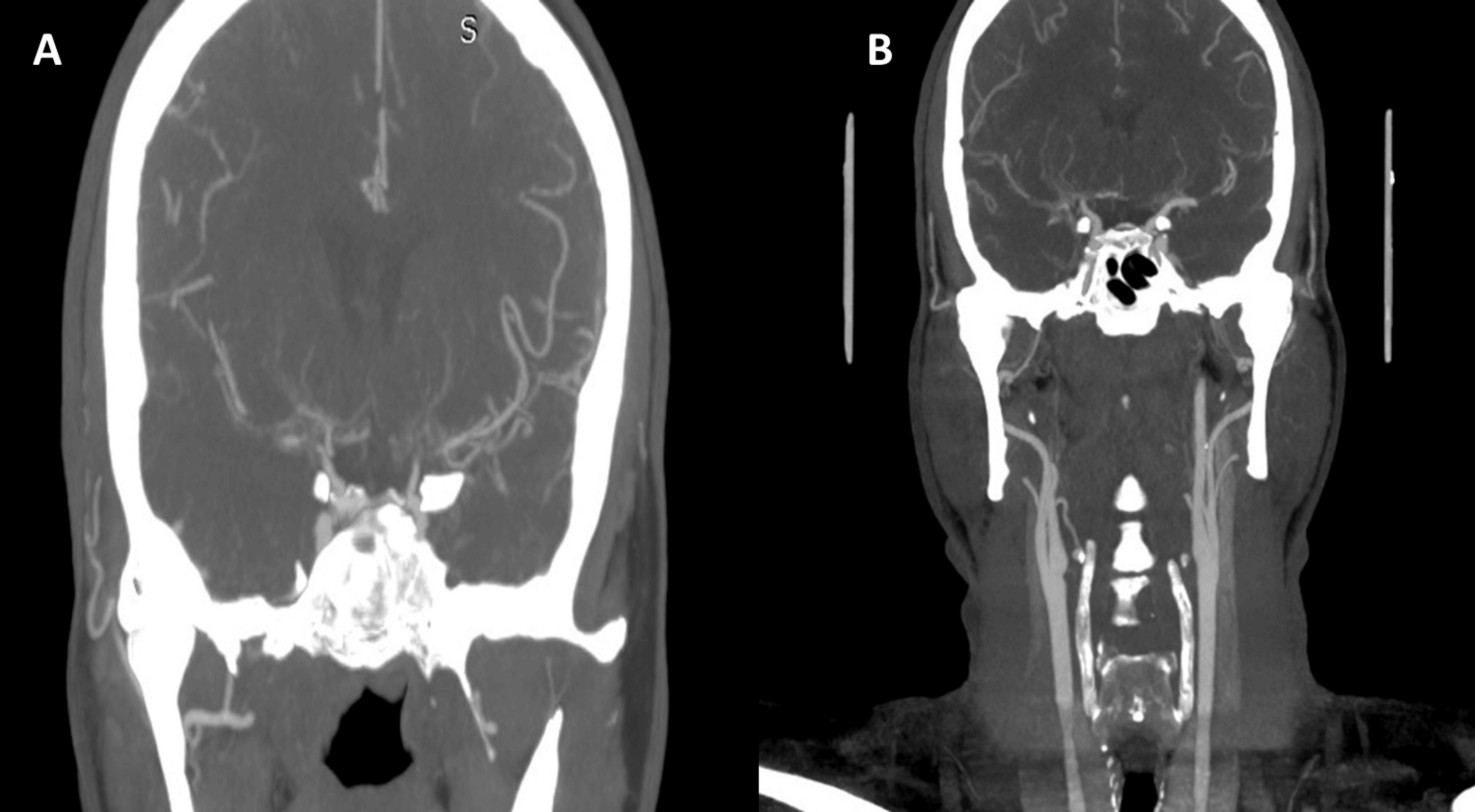
CT angiography: (A, B) irregular stenosis of the distal portion of the right ICA CT: computed tomography, ICA: internal carotid artery

A 45-year-old woman, followed for years for hyperthyroidism due to a toxic multinodular goiter and on synthetic antithyroid medication, consulted the emergency department for left weakness accompanied by facial asymmetry, occurring two days after a normal state. Neurological examination revealed left central facial paralysis and left hemiparesis. The National Institutes of Health Stroke Scale score was 7, and capillary glucose and blood pressure were normal. Cerebral CT showed right fronto-parietal hypodensity of ischemic appearance (Figure 8), and CT angiography revealed occlusion of the M2 segment of the right middle cerebral artery (Figure 9). Digital subtraction angiography revealed subocclusive stenoses of the carotid arteries and the M1 and A1 segments bilaterally, with the development of an abnormal vascular network of medial lenticulostriate arteries bilaterally, taking up the distal M1 segments in a weak current. Thyroid function tests revealed hyperthyroidism (TSH 0.05 IU/ml, FT4 5.1 ng/dL, FT3 7.61 pg/mL) (Table 1). Thyroid ultrasound showed an enlarged gland with nodules of varying sizes, and immunological and CSF tests showed no abnormalities. Due to the persistence of thyroid disorders and the size of the goiter, total thyroidectomy was performed, followed by lifelong replacement therapy. Thyroid levels normalized, and the patient had no ischemic or hemorrhagic recurrence after surgery.

## Discussion

Moyamoya disease and syndrome involve a progressive narrowing and eventual blockage of the distal segments of the internal carotid arteries and their major branches. While the condition typically presents bilaterally, some cases are limited to one hemisphere, referred to as unilateral or probable Moyamoya. The most widely accepted definition of Moyamoya disease describes it as an idiopathic bilateral occlusion affecting the vessels of the circle of Willis. This definition emphasizes the key aspects of the condition: its anatomical location (the circle of Willis), the underlying mechanism (vascular occlusion), its typical bilateral manifestation, and the absence of a known cause (idiopathic) [2].

Genetic factors of Moyamoya disease

Recent research has led to a better understanding of the genetic and environmental factors involved in Moyamoya disease. The discovery of a genetic locus strongly associated with the disease in the ring protein gene 213 supports the existence of weak areas in the human genome that lead to susceptibility to Moyamoya disease [3]. Beyond genetic predisposition, several environmental contributors have been considered in the etiology of Moyamoya disease. Among these are infectious triggers, immune system involvement, including cellular responses and autoantibody production, as well as localized hemodynamic stress on vulnerable vascular regions [4,5].

Associated diseases of Moyamoya syndrome

Digital subtraction angiography findings typical of Moyamoya disease, namely stenosis/occlusion of distal ICAs and development of basal collaterals, are not specific to this disease. Thus, we will use the provisional term Moyamoya vasculopathy to refer to this phenomenon. Occasionally, Moyamoya vasculopathy develops in patients with other well-characterized diseases or syndromes. However, clinicians have noted that the following diseases are associated with Moyamoya vasculopathy. These include neurofibromatosis type 1, Down's syndrome, thyroid pathologies, systemic diseases, therapeutic irradiation, and sickle cell anemia, among others.

Our fourth case illustrates the association of Moyamoya syndrome with thyroid dysfunction, which has been rarely reported. Ohba et al. reviewed reports of 31 patients with both Graves' disease and intracranial stenosing-occlusive disease published in the literature [6,7]. The majority of patients were female, with a mean age of 29.3 years. All presented with cerebral infarctions or transient ischemic attacks, and none exhibited hemorrhagic symptoms. All except two individuals were found to be in a thyrotoxic state at the time of their ischemic episodes, supporting the hypothesis that elevated thyroid hormone levels may contribute to vascular dysfunction by enhancing sensitivity to sympathetic stimulation. In cases of unmanaged thyroid disorders, the hyperdynamic circulatory state associated with thyrotoxicosis may precipitate ischemic strokes, particularly when chronic vascular changes have already compromised cerebrovascular reserve. While the most effective therapeutic approach for thyroid-associated Moyamoya syndrome remains uncertain, proper management of thyroid function can mitigate hemodynamic stress and lower the risk of ischemic complications.

Nevertheless, when imaging demonstrates impaired cerebral perfusion, revascularization surgery is recommended, especially given the heightened risk of recurrence in cases of thyrotoxicosis relapse [6]. Moyamoya syndrome has also been linked to several autoimmune disorders, including antiphospholipid syndrome, systemic lupus erythematosus, ulcerative colitis, and, less commonly, Sjögren's syndrome [8-10]. Immunohistochemical analyses have, in some instances, identified immunoglobulin deposits within the intima of the internal carotid artery [11] and subendothelial infiltration by T lymphocytes [12], suggesting an immune-mediated component in the pathogenesis of the condition.

Challenges of diagnostic imaging

The diagnosis of Moyamoya disease and syndrome is based on anatomical and functional aspects. First-line imaging examinations include cerebral angiography scan and MRI angiography to identify anatomical features, such as stenosing-occlusive changes and collateral vessel formation. A more recently described sign is the "Ivy sign" on the FLAIR sequence, corresponding to prominent leptomeningeal collaterals, which result in high signal on FLAIR due to slow flow and vivid contrast enhancement on post-contrast T1. Digital subtraction angiography remains the gold standard for confirming the diagnosis, the stage of the disease, and the search for microaneurysms, a source of cerebral hemorrhage. Indeed, the definitive diagnosis of Moyamoya disease is always based on the results of digital subtraction angiography, which defines the dynamic vascular changes and enables the disease to be classified according to systems such as the Suzuki classification [13]. Although the new RCMD recommendations also include the use of MRI and MRA in some instances [14].

Treatment strategy

Although there is currently no specific therapeutic strategy to prevent or reverse the vascular anomalies associated with Moyamoya disease and syndrome, the interventions used for stroke prophylaxis have probably altered the natural course of the disease. Treatments can be categorized into two main types: conservative and interventional. The clinical approach is primarily based on symptomatic and prophylactic treatment, including the use of antiplatelet agents. Acetylsalicylic acid is strongly recommended to prevent the recurrence of ischemic attacks, with clopidogrel as an alternative. In addition, strict control of additional risk factors, such as dyslipidemia, hypertension, and diabetes, is strongly recommended. Surgical treatment aims to minimize cerebral ischemia by improving cerebral blood flow and reducing the hemodynamic stress responsible for cerebral hemorrhage, thereby reducing the recurrence rate of ischemic attacks and producing significantly more favorable clinical outcomes than those achieved with conservative treatment [13,14].

In symptomatic patients in the United States, conservative (non-surgical) management is associated with a high risk of recurrent events, with a five-year risk of recurrent ischemic events reaching up to 65% in patients with unilateral disease and 82% in those with bilateral involvement. Furthermore, recurrent hemorrhages have been reported in Japanese populations, occurring in approximately 30% to 60% of patients during long-term follow-up [15-17].

Surgical revascularization utilizes branches of the external carotid artery and can be categorized into two types: direct and indirect. Direct anastomosis results in an immediate increase in cerebral perfusion and should therefore be considered the first-line treatment [15]. When this is not possible, indirect methods are useful and technically easier to perform, although they require more time to restore blood flow adequately. Combinations of direct and indirect bypass surgery are also available [18]. The true benefit of revascularization for adults with asymptomatic Moyamoya disease remains unknown, and there is as yet no consensus on the necessity or timing of surgery in these patients [15]. Endovascular treatment of ischemic Moyamoya with stents or angioplasty has a low success rate (25%) and high complication rates, mainly due to complete vessel wall narrowing. In cases of hemorrhage related to pseudoaneurysms, distal parent vessel occlusion is effective, with success rates of 80% for endovascular treatment or revascularization surgery [19]. After surgery, long-term clinical and imaging follow-up is necessary to ensure the effectiveness of the procedures. Postoperative collateral formation can be assessed by cerebral angiography and graded according to the Matsushima grading scale [20].

## Conclusions

Moyamoya disease and syndrome, although a rare pathology, remains a diagnostic and therapeutic challenge, particularly when secondary to other systemic pathologies. Early diagnosis and appropriate management of the underlying cause are crucial for improving patients' functional prognosis. Advances in neuroimaging and surgery provide new insights for a deeper understanding and more effective treatment of this complex disease.
